# Sex-dependent impact of early-life stress and adult immobilization in the attribution of incentive salience in rats

**DOI:** 10.1371/journal.pone.0190044

**Published:** 2018-01-11

**Authors:** Silvia Fuentes, Javier Carrasco, Abigail Hatto, Juan Navarro, Antonio Armario, Manel Monsonet, Jordi Ortiz, Roser Nadal

**Affiliations:** 1 Institut de Neurociències, Universitat Autònoma de Barcelona, Barcelona, Spain; 2 Psychobiology Unit (School of Psychology), Universitat Autònoma de Barcelona, Barcelona, Spain; 3 Animal Physiology Unit (School of Biosciences), Universitat Autònoma de Barcelona, Barcelona, Spain; 4 Biochemistry Unit (School of Medicine), Universitat Autònoma de Barcelona, Barcelona, Spain; 5 CIBERSAM, Instituto de Salud Carlos III, Barcelona, Spain; Technion Israel Institute of Technology, ISRAEL

## Abstract

Early life stress (ELS) induces long-term effects in later functioning and interacts with further exposure to other stressors in adulthood to shape our responsiveness to reward-related cues. The attribution of incentive salience to food-related cues may be modulated by previous and current exposures to stressors in a sex-dependent manner. We hypothesized from human data that exposure to a traumatic (severe) adult stressor will decrease the attribution of incentive salience to reward-associated cues, especially in females, because these effects are modulated by previous ELS. To study these factors in Long-Evans rats, we used as an ELS model of restriction of nesting material and concurrently evaluated maternal care. In adulthood, the offspring of both sexes were exposed to acute immobilization (IMO), and several days after, a Pavlovian conditioning procedure was used to assess the incentive salience of food-related cues. Some rats developed more attraction to the cue predictive of reward (sign-tracking) and others were attracted to the location of the reward itself, the food-magazine (goal-tracking). Several dopaminergic markers were evaluated by in situ hybridization. The results showed that ELS increased maternal care and decreased body weight gain (only in females). Regarding incentive salience, in absolute control animals, females presented slightly greater sign-tracking behavior than males. Non-ELS male rats exposed to IMO showed a bias towards goal-tracking, whereas in females, IMO produced a bias towards sign-tracking. Animals of both sexes not exposed to IMO displayed an intermediate phenotype. ELS in IMO-treated females was able to reduce sign-tracking and decrease tyrosine hydroxylase expression in the ventral tegmental area and dopamine D1 receptor expression in the accumbens shell. Although the predicted greater decrease in females in sign-tracking after IMO exposure was not corroborated by the data, the results highlight the idea that sex is an important factor in the study of the long-term impact of early and adult stressors.

## Introduction

It has been well-established that early-life stress (ELS) induces profound long-term effects in adult functioning. For instance, several findings in humans indicate that ELS decreases adult responses to reward [1–3]. Recent conceptualizations highlight the fact that both negative consequences and increased resilience may be found after ELS for several reasons [4]. The question of how the exposure to ELS interacts with other stressors experienced in adulthood has important translational value. There are several possibilities for this interaction. The “two-hit” [5, 6] and “cumulative stress” [7, 8] hypotheses propose that the accumulation of early and adult exposure to stress compromises the ability of the subject to cope with stress, thus inducing “detrimental” effects. On the other hand, the “match–mismatch” models predict that after an ELS experience, individuals will show improvements in coping behavior and adaptability to further exposures to stressors in adulthood [9, 10]. This interaction may be potentially relevant when subjects are exposed to severe (traumatic) stressors.

Although findings are not fully consistent, patients with post-traumatic stress disorder (PTSD) seem to present a blunted response to reward, affecting both the motivational and the consummatory phases of reward [11]. Sex-dependent effects in PTSD symptoms have also been reported, with anhedonia-related symptoms higher in women than men [12, 13]. At least under some circumstances, prior exposure to early adversity may worsen PTSD symptomatology (for a review, see [14]). To our knowledge, possible sex-dependent differences in the attribution of incentive salience to reward-related cues have not been specifically addressed in adult PTSD patients exposed to ELS.

In rodents, adult acute exposure to severe stressors may induce a range of long-term effects in behavior/cognition and the central nervous system [15], and some of these changes may be reminiscent of those seen in PTSD. Our laboratory has been studying the effects of exposure to immobilization stress on boards (IMO) in recent years. As indicated by pituitary-adrenal hormones and other biological markers, IMO is more intense than other stressors such as footshock [16]. Our previous results in male rats indicate that a single IMO induces impaired spatial memory on the Morris water maze [17, 18], fear extinction [19], reduced intake of sweet solutions as a measure of anhedonia [20], increased acoustic startle response [21], and sensitization of the hypothalamic-pituitary-adrenal axis to further heterotypic stressors [22], which are changes that mimic some of those reported in PTSD patients [23]. Few data are available regarding the sex-dependent effects of IMO exposure, but at least some of the long-term effects of IMO may be sex-dependent [24]. The study of sex differences on the impact of severe stressors in animal models [25] has important functional implications because, in humans, the prevalence of PTSD is higher in females than males [26], and gender differences in symptom expression have also been described [12, 13].

Learning how to predict changes in the environment is a fundamental need in human and animal behavior. In this context, the attribution of incentive salience to reward-related cues is usually adaptive, promotes survival and guides decision-making processes [27]. However, in pathological circumstances, an increase in the motivational value of those cues may lead to compulsive over-eating or drug-seeking behaviors [28], and failure to appropriately assign incentive salience may be related to amotivation/anergia and anhedonia, which are symptoms of several neuropsychiatric diseases, such as schizophrenia [29], depression [30] and PTSD [11]. Strong individual differences in the ability to attribute incentive salience to reward-related cues has been described in animal models [28, 31] and humans [32–34].

The attribution of incentive salience (motivational value) to reward-associated cues has been widely studied through a Pavlovian conditioning model (see [28] for a review). There are some animals (“sign-trackers”, ST) that are more attracted to the cues that signal the reward than to the location where the proper reward is delivered (“goal-trackers”, GT). ST and GT behavior neural substrates are partially dissociated. Dopamine is involved in the attribution of incentive salience and some studies have detected differences between ST and GT animals in key dopaminergic markers [35–36]. An optimal tone of dopamine activation is required for the processing of salient cues, and ST behavior seems to be more sensitive to dopaminergic transmission than GT behavior [37–39]. However, to our knowledge, there are no previous studies in animal models regarding how the attribution of incentive salience to reward-related cues is modified by previous ELS and exposure to traumatic adult stressors, and how these changes are related to putative changes in dopaminergic markers.

Given all the above, our aims are to study (i) the long-term effects of ELS in the attribution of incentive salience to reward-related cues, (ii) how ELS experience modifies the long-term impact of an additional severe stressor in adulthood (IMO), (iii) the role of sex in these effects and (iv) the relationship between incentive salience changes and some key dopaminergic markers. As a model of ELS, an adaptation of the one proposed by Baram and collaborators was used [40] that consists of the restriction of nesting material to the dams and pups during the first postnatal days. Due to the fact that maternal care may buffer some of the negative consequences of ELS, we evaluated maternal behavior during and after the restriction of nesting material [41–43]. Given the possible blunted response to reward in PTSD patients and the presence of more anhedonia-related symptoms in women, our hypothesis was that IMO will decrease, especially in females, the attribution of incentive salience to food-related cues with a differential impact as a function of previous ELS experience.

## Materials and methods

### Animals

Long-Evans (RjOrl:LE) rats were always housed in Makrolon transparent polycarbonate wire-topped cages with a solid bottom (26.5 x 42.5 x 18.5 cm, Ref. 1291 Eurostandard Type III H) containing sawdust bedding (Lignocel 3/4, Harlan) in a climate-controlled environment at 20–21°C on a 12-hour light–dark cycle (lights on 8:00 am). During gestation and lactation, rats were fed with a high protein diet (Ref. 2918 Teklad Global 18% protein, Harlan), and after weaning, they were fed a regular diet (Ref. 2014C Teklad Global 14% protein, Harlan). Rats were allowed ad libitum access to food except when the autoshaping procedure started. Rats always had free access to filtered tap water. No specific environmental enrichment program was used in the animal facility. All animal protocols were in accordance with the European Communities Council Directive 2010/63/EU and the Spanish legislation (RD53/2013) and were approved by the Ethics Committee for Human and Animal Research of the Universitat Autònoma de Barcelona, and by the Catalan Government (Generalitat de Catalunya). A maximal effort was expended to minimize the suffering of the animals and the number of animals used. The animal’s health was monitored daily.

### Early treatment and maternal behavior

**Fig 1:**
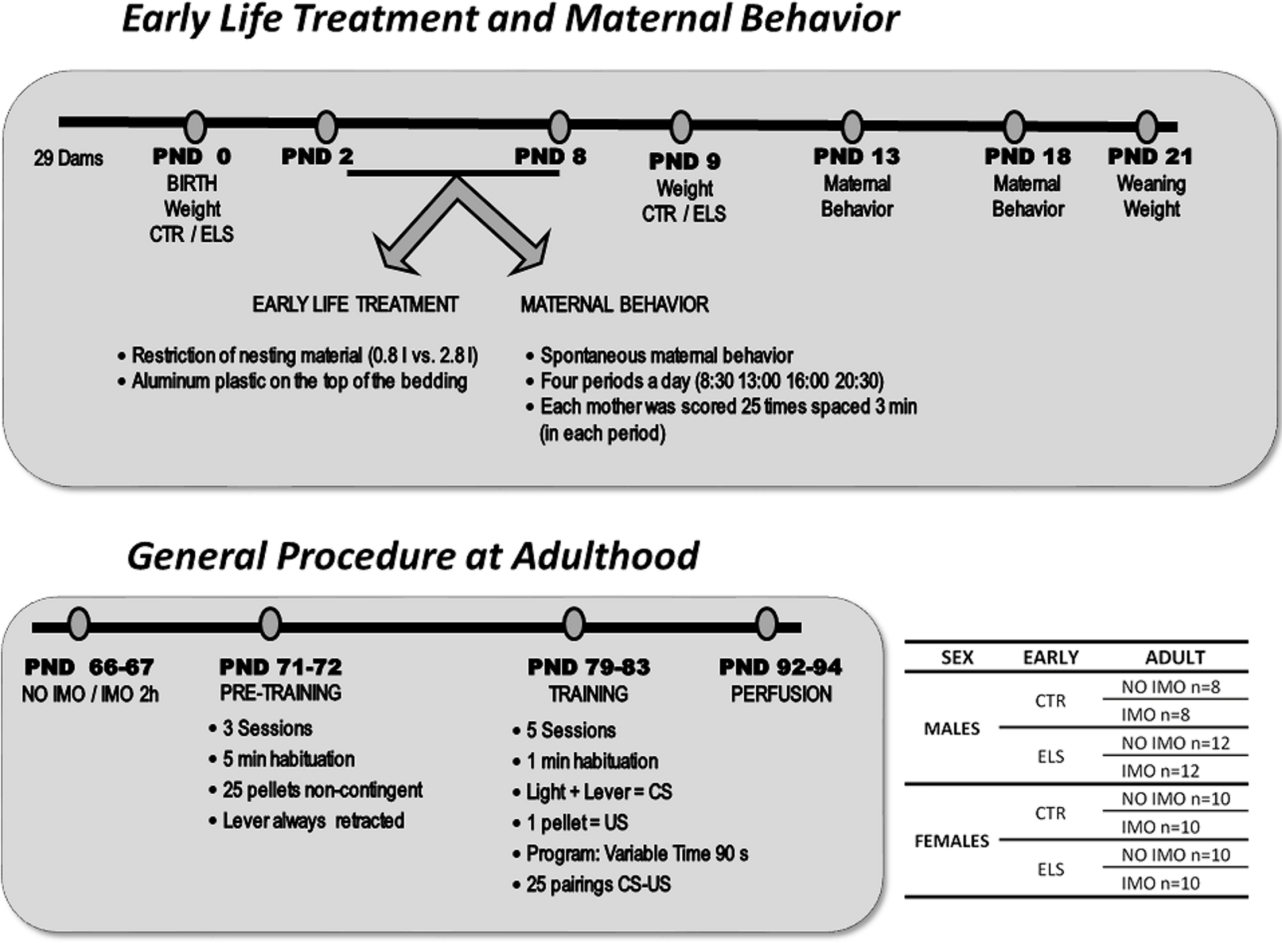
Summary of the experimental procedure. CS: conditioned stimulus; CTR: control; ELS: early-life stress; IMO: immobilization; PND: postnatal day; US: unconditioned stimulus.

A summary of the experimental procedure is shown in Fig 1. Mated programmed dams arrived from Janvier (France) at GD 15. For the mating, each dam (approximately 8 weeks of age, primiparous) was paired with a different male (1 male / 1 female) for one night. In the beginning, we used 29 different dams. All the dams were in the same vivarium, with no other experiments going on, and special care was taken to restrict access to the room. Dams were inspected twice daily for delivery (at 8:00 and at 15:00 h). The day of delivery was PND 0. Pups were counted, sexed, the litter cut to 12 if higher (maintaining, if possible, a sex ratio between 0.4–0.6) and weighed (as a whole for each dam, separated by sex). Cross-fostering was never done. Births were scattered across 2 days. On PND 2, dams were divided at random into 2 groups: control (CTR, non-ELS) and ELS. The ELS consisted of restricting nesting material by giving access to a limited amount of bedding (0.8 l instead of the 2.8 l that were given to CTR animals), and a plastic wire (0.5 x 0.5 cm) was placed on the top of the bedding of the ELS rats. A paper towel (20.5 x 20 cm) was provided to all dams on PND 2 and again on PND 5. The bedding was not changed until PND 9 when ELS dams were returned to CTR conditions, and all pups were weighed (as a whole for each dam, separated by sex).

Spontaneous maternal behavior during PND 2 to 8 was measured 4 times a day (8:30, 13:00, 16:00 and 20:30 h) following an adaptation of our previous studies [41, 44]. Maternal behavior was also measured on PND 13 and PND 18, which was 5 and 10 days after the treatment finished. The measures were taken on-line by an observer that remained quiet in the room. Within each observation period, the behavior of each mother was scored 25 times spaced 3 min apart (25 observations × 4 periods per day × 7 days = 700 observations/mother). The following behaviors were scored as present or absent: (1) mother licking-grooming (body+anogenital region) any pup, (2) mother nursing pups in an arched-back posture with rigid limbs (“high kyphosis”), (3) mother nursing in a “blanket” posture in which the mother just lies over the pups (“prone nursing”) or the mother’s limbs are rigid but maintain a low dorsal arch posture (“low/partial kyphosis”), (4) mothers nursing in a “passive” posture (“supine nursing”) in which the mother lies on her back or side while the pups are nursing, and (5) mother “off” nest (no maternal contact). Other behavioral measures were taken (mother retrieving pups and mother preparing a nest), but the frequency of these patterns was very low.

The bedding was changed again on PND 16, and pups were weaned on PND 21. After weaning, they were housed by sex in groups of 4 (each one from a different mother) and kept undisturbed until PND 60. On this day, same sex rats were housed in pairs and individually weighed. In each one of the 8 groups (see below), only 1–2 pups from the same mother were used. Before initiating adult testing, rats were handled at least for 3 days.

### General procedure in adulthood

Animals were exposed to IMO stress or remained undisturbed at PND 67–68. IMO [45, 46] was conducted by taping their four limbs to metal mounts attached to a wooden board for 2 h. Head movements were restricted with two plastic pieces (7 x 6 cm) placed on each side of the head, and the body was secured to the board by means of a piece of plastic cloth (10 cm wide) attached with *Velcro*^R^ that surrounded the trunk.

Thus, a total of 8 groups were established, divided by sex (M/F) and treatments (selected at random), early treatment (CTR/ELS) and adult treatment (NoIMO/IMO): M-CTR-NoIMO (n = 8), M-CTR-IMO (n = 8), M-ELS-NoIMO (n = 12), M-ELS-IMO (n = 12), F-CTR-NoIMO (n = 10), F-CTR-IMO (n = 10), F-ELS-NoIMO (n = 10), F-ELS-IMO (n = 10). Magazine pretraining (see below) started 5 days later, and the proper autoshaping procedure started 6 days after the magazine pretraining finished. Estrous cycle was not monitored, but female approach behavior towards the lever or the magazine has not been found to differ across the cycle [47]. The order of the experimental groups tested was randomized across the session.

### Incentive salience boxes

The task was conducted in 8 standard operant boxes (Panlab-Harvard, LE1005, Barcelona, Spain). Each chamber (25 x 25 x 25 cm) had a clear Plexiglas door and black aluminum sidewalls. The floor was composed of 19 stainless steel rods (3 mm in diameter), spaced 1 cm center to center. A house-light (4 cm diameter 2.4-W, 24-V) was placed in the right wall 22 cm from the floor. In the left wall, two metal retractable response levers were placed (6 cm above the floor, 1.8 cm wide and 3 cm long, 7.5 cm from the midline) on either side of a food magazine (3.5 x 3.5 cm) provided with an electromagnetic detector. Placed above each lever and above the food magazine (12.5 cm from the center of the light to the floor) was another light (3 lights more in total). The force needed to press the lever was 18–20 g. The software (Packwin 2.0.03, Panlab-Harvard) controlled the administration of the different stimuli and recorded the data (number of lever presses and entries inside the food magazine and latencies of these behaviors). The chambers were inside a metallic sound attenuating box (67 x 53 x 55 cm) provided with a fan to mitigate strange sounds. Behavior was also monitored by means of a miniature camera (Ref. KPC-S500P3, KT&C co.) mounted on the front panel and stored in a JVC VR-716 digital video recorder. Bio-Serv sucrose pellets (45 mg, Ref F0042, Frenchtown, NJ, USA) were used as a reinforcer. The chambers were carefully cleaned with a solution containing soap between rats.

### Incentive salience procedure

To habituate the animals to the taste of the pellets and reduce neophobia, 25 pellets were introduced inside the home-cage over 2 consecutive days prior to any training. Later, rats were exposed to a pretraining (3 sessions. Friday, Monday and Tuesday) for 5 min after being introduced inside the box, 25 food pellets were non-contingently delivered with a variable time 30 s schedule (random between 1–60 s, 1 pellet each time). These sessions lasted approximately 17.5 min, and the levers were always retracted. Animals were food-restricted until approximately 87% of the body-weight (taking as a reference the weight at 8 days post-IMO) and were always fed at least for 1 h after the end of the session.

After the pretraining, the proper autoshaping training started (adapted from [48]). A one-lever non-discriminative procedure was used. Animals were exposed to 5 consecutive sessions (1 daily, Monday to Friday). One min after the animal was introduced inside the operant box, the first trial started signaled by the illumination of the house-light, which remained ON during the whole session. Each trial consisted of the illumination of the lever-light and the insertion of that lever (which act as a compound conditioned stimulus, CS) into the box for 8 s. For half of the animals, the right lever/light was used, and the left lever/light was used for the remaining half. Only one lever was available during the session, and the other one remained retracted the whole time. Immediately after the retraction of the lever, one food pellet (which acted as an unconditioned stimulus, US) was delivered inside the food magazine. The delivery of the pellet was independent of the behavior of the animal and followed a variable time 90 s program (range between 30–150 s). The total number of trials during a session was 25. The session duration was approximately 37.5 min. Thus, the delivery of the pellet was always paired to the previous appearance of the lever and the illumination of the lever-light. A non-paired group was not included because previous studies showed that animals receiving pseudorandom pairing of the CS and the US do not develop a conditioned response [35]. The first day of autoshaping animals were 81–82 days old (14 days post-IMO).

### Incentive salience measures

The software provided data about the number of entries inside the food magazine (during lever presentation, during pellet delivery and during the inter-trial interval, ITI). From the video recordings, additional measures were taken for days 1 and 5 by means of the Observer software (Noldus, The Netherlands, version XT 11). An observer blind to the treatment analyzed the ST and GT behavior. ST behavior was measured by the time spent in behaviors towards the lever including “approach” (“preparatory”) behaviors without physical contact (sniffing) and “contact” (“consummatory”) behaviors (touching/grasping, biting/nibbling). GT behavior was measured by the time spent inside the food magazine during lever presentation. The reliability of these observations was assessed by the analysis of the duration of behavior towards the lever (day 5) by two independent observers, and the correlation between those measures was of r(78) = + 0.975, p < 0.001. The number of contacts with the lever-light were also measured but the frequency of this behavior was very low. On day 5, an additional measure (ST-GT score) was included to analyze the behavior towards the lever and towards the magazine (during lever presentation) at the same time using this formula: percent of time spent in behavior towards the lever (with regard to the possible 400 s of interaction: 25 trials x 8 s of duration of each trial)–percent of time spent in behavior towards the magazine). Thus, a positive score indicated that the animals interacted more with the lever than with the magazine, and a negative score indicated the opposite. Orienting behavior towards the lever (moving the body towards the lever in the moment it appeared, without physical contact and without sniffing it) was also measured for days 1 and 5. To analyze motor activity, the movements of the animal inside the chamber were measured by videotracking (Smart software, Panlab-Harvard, version 2.5.21).

### In situ hybridization

Eight animals from each subgroup were selected at random to analyze the expression of some dopaminergic markers. On PND 92–94, rats were anesthetized with isoflurane and perfused with saline solution (4°C) for 2 min and with 4% paraformaldehyde and 3.8% borax (4°C) for 8–10 min. Then, brains were removed, post-fixed overnight at 4°C and cryoprotected the next day [0.2 M NaCl, 43 mM potassium phosphate (KPBS) containing 30% sucrose] for 48 h at 4°C. Next, each brain was frozen on dry ice, and six series of 14-μm coronal sections were obtained with a cryostat (Frigocut 2800; Leica, Nussloch, Germany) and stored at − 20°C in an anti-freeze solution (30% ethylene glycol, 20% glycerol in 0.25 mM phosphate buffer at pH 7.3).

Following Paxinos and Watson atlas [49], serial coronal sections (14 μm thick) through the dorsolateral striatum (DLST)-dorsomedial striatum (DMST) and the nucleus accumbens shell (AcbS) (Bregma 1.2 mm to 0.7 mm), the ventral tegmental area (VTA) and the substantia nigra pars compacta (SNpc) (Bregma -5.3 mm to -5.8 mm) and the locus coeruleus (LC) (Bregma -9.68 mm to -10.04 mm) were mounted onto Superfrost Plus slides (Thermo scientific; Menzel-Gläser, Braunschwieg), which were then maintained at − 80°C until the day of analysis. See S1 Fig for the size of the areas analyzed.

Tyrosine hydroxylase (TH) mRNA was measured within the LC, VTA and SNpc. D1 dopamine receptor mRNA was measured within the DLST, DMST and AcbS. The TH probe was generated from a pBluescript SK plasmid containing a fragment of 1.7 kb of rat TH cDNA. The cDNA was then subcloned into a pGEM-4Z (Promega) plasmid. The D1 probe was generated from a HindII-SacI-3 genomic fragment of 2.4 kb of rat DNA coding for D1, within plasmid pGEM-3. Plasmids were linearized, and radioactive antisense cRNA copies were generated using a transcription kit (Roche, Germany) in the presence of [α-35S]-UTP (specific activity >1000 Ci/mmol, Perkin Elmer, Spain). The labeled cRNA was purified using Mini Quick spin RNA columns (Roche, Spain) and stored at -20°C.

The protocol used was adapted from [50] and used in our previous studies (e.g., [51, 52]). All solutions were pretreated with DEPC and sterilized before use. Sections were post-fixed in 4% PFA + Borax, rinsed in KPBS, digested with 0.01 mg/ml proteinase K (Roche Diagnostic, Germany), rinsed in DEPC-treated water and 0.1 M triethanolamine pH 8.0 (TEA; Sigma) and acetylated in 0.25% acetic anhydride in 0.01 M TEA, washed in 2 × SCC, dehydrated through graded concentrations of ethanol and air-dried. Thereafter, 100 μl of hybridization solution (50% formamide, NaCl 0.3 M, Tris–HCl 10 mM pH 8.0, EDTA 1 mM pH 8.0, 1 × Denhardt's solution, 10% dextran sulfate, yeast tRNA 500 g/ml and 10 mM DTT) containing 1 × 10^6^ dpm of the labeled probe (approx. 1 pMolar) was spotted onto each slide and sealed with a coverslip. After a 16–18 h incubation in a humid chamber at 60°C, the slides were washed in descending concentrations of SSC containing 1 mM DTT (Sigma, Spain), including one wash at 60°C, digested with RNase A (0.02 mg/ml, Roche, Spain), dehydrated through a series of ethanol solutions and air-dried. The slides were then exposed to autoradiography film (XAR-5 Kodak Biomax MR, Kodak, Spain) for 14 h for TH and 48 h for D1. All the slides to be compared were exposed in the same cassette.

Densitometric analyses were performed on the autoradiography films. The mRNA levels were semi-quantitatively determined in both hemispheres in 3–4 sections per brain area and per animal. The sections to be analyzed were photographed under a 4 × microscope objective (Eclipse E400, Nikon, Japan) with a DXM1200 digital camera (Nikon, Japan). The illumination conditions, exposure time, sensitivity to light and resolution conditions were kept constant across all the photographs to be compared. Photomicrographs were subsequently quantified in a blind manner with Scion Image software (W. Rasband, NIH, USA; available on the web at http://rsb.info.nih.gov/nih-image or http://www.scioncorp.com). The program uses an 8-bit scale for gray values, with 0 indicating absolutely white and 255 absolutely black. For each brain area, a threshold for gray value was selected, and the pixels with values under these thresholds were not further considered in the analysis. Thresholds were chosen in a way that retained the specific signal and removed most of the background. Once the optimal threshold was determined, it was kept constant across all the subjects to be compared. The mRNA expression was estimated from representative pictures in regions of interest (ROIs) of the same size for all animals and for each particular nucleus of interest, determining the gray levels of the pixels above the background and the number of them. The outcome of the quantification software were the mean gray level of the selected pixels (intensity), the number of the selected pixels (area) and the integrated density (area x intensity) refereed in arbitrary units. In all cases, the intensity of the signal was within the linear range as evaluated by comparison with ^14^C microscales (GE Healthcare, UK).

### Statistical analysis

Data were analyzed with the Statistical Program for Social Sciences, SPSS (version 24). Maternal behavior was analyzed by means of a general linear model (GLM) with one between-subject factor of early treatment (two levels, CTR and ELS) and one within-subject factor (DAY or HOUR, seven or four levels, respectively). For adult data, different GLMs were used with three between-subject factors: sex (two levels, males and females), early treatment, ELS (two levels, CTR and ELS) and adult IMO stress (two levels, NO-IMO, IMO). To achieve homogeneity of variance, if needed, log-transformations were made. If log-transformations were not useful (because the data had several zeros or because even after the transformation the data did not achieve homogeneity of variance), a generalized linear model [53] instead of a GLM was performed. As it was predicted that the IMO treatment would have a differential, sex-dependent impact on the function of previous ELS, the IMO x SEX x ELS interaction was further analyzed, and a priori planned contrast was performed in the IMO-treated rats comparing ELS and non-ELS animals in each one of the sexes. The Pearson correlation coefficient (two-tailed) was used to correlate behavioral and ISH data. Data are given as the mean ± SEM.

## Results

### Early life treatment and maternal behavior

One dam from each group was excluded from the study because the pups died, giving a total of 27 dams. Births were scattered across 2 days. The number of pups (at birth and after adjustment) and sex ratio at PND 0, PND 9 and at weaning was not affected by the early life treatment. From all the pups weaned, 80 pups were selected at random to be used in this experiment.

On PND 0 and on PND 9, pups of the same sex were weighed as a whole for each dam. No differences in body weight on PND 0 were observed. On PND 9 (after the ELS treatment), no statistically significant differences were found in the body weight of male pups between CTR and ELS groups (18.31 ± 0.62 g versus 17.08 ± 0.42 g, respectively; t(25) = 1.65, p = 0.11). In contrast, the early life treatment decreased body weight in female rats (CTR: 17.46 ± 0.55 g versus ELS: 15.91 ± 0.42 g, t(25) = 2.26, p < 0.05). On PND 21 and PND 60, pups were weighed individually and no between-group differences related to early life treatment were found. Males were heavier than females, as expected (SEX at PND 21: F(1, 72) = 13.31, p < 0.001; SEX at PND 60: F(1, 72) = 615.85, p < 0.001; all the other factors and interactions were NS: ELS, IMO, ELS x IMO, ELS x SEX, IMO x SEX, IMO x SEX x ELS).

**Fig 2:**
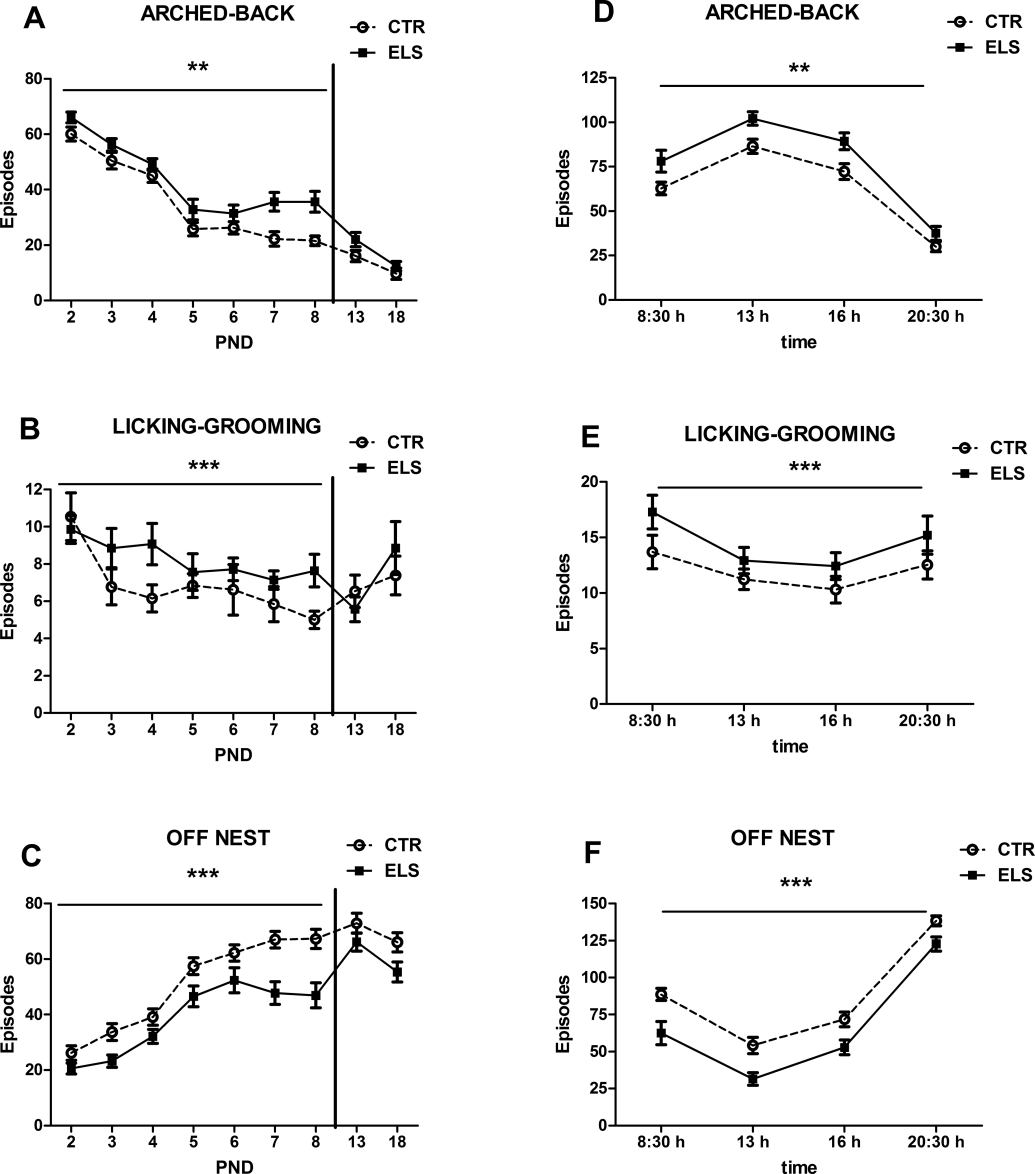
Maternal behavior measures. Left panels: Number of episodes of arched-back (A), licking-grooming (B) and “off” nest (C) behaviors during postnatal days (PND) 2 to 8, and on PND 13 and PND 18 (sum of all the times of the day analyzed). Right panels: Number of episodes of licking-grooming (D), arched-back (E) and off-nest (F) behaviors at 4 different times of day (sum of PND 2 to 8). The results are shown for control (CTR) and early-life stress (ELS) dams. ** p < 0.01 and *** p < 0.001 versus CTR. Means and SEM are shown.

**Fig 3:**
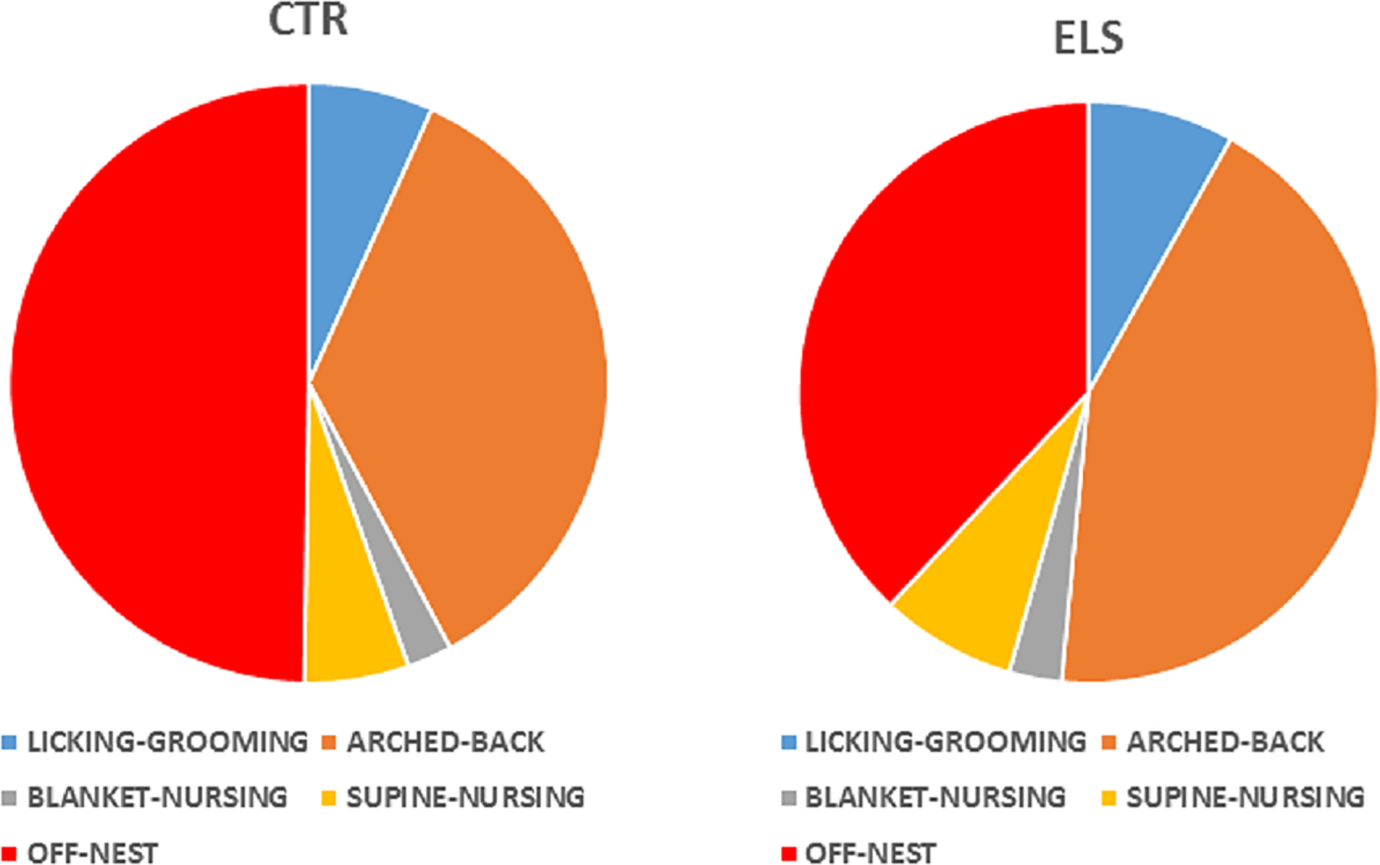
Types of maternal behavior. Distribution of different types of maternal behavior in control (CTR) and early-life stress (ELS) dams during PND 2 to 8.

Maternal behavior was analyzed for each day (the 4 measures on each day were collapsed) and for each time period (all days pooled for each time period). Arched-back behavior (Fig 2A) from PND 2 to 8 decreased across days, and early stress treatment increased this measure globally [ELS: F(1,25) = 10.47, p < 0.01, DAY: F(6, 150) = 79.99, p < 0.001, ELS x DAY: NS]. Similar results were obtained with licking-grooming [Fig 2B, ELS: F(1,25) = 13.21, p = 0.001, DAY: F(6, 150) = 3.04, p < 0.01, ELS x DAY: NS]. The number of episodes where the dam was “off” nest (Fig 2C) increased across the days, and early stress treatment decreased this measure globally [ELS: F(1,25) = 16.25, p < 0.001, DAY: F(6, 150) = 58.33, p < 0.001, ELS x DAY: NS]. When the restriction of bedding material was finished, maternal behavior normalized (PND 13 and PND 18, all factors NS in all cases). The effects of ELS on arched-back, licking-grooming and “off” nest behaviors were maintained for all periods analyzed (Fig 2D, 2E and 2F, respectively, HOUR x ELS: NS in all cases). With regard to other maternal measures (blanket-nursing and supine-nursing), no between-group differences were detected. A summary of the pattern of maternal behaviors exhibited by each group is presented in Fig 3.

### Incentive salience

**Fig 4:**
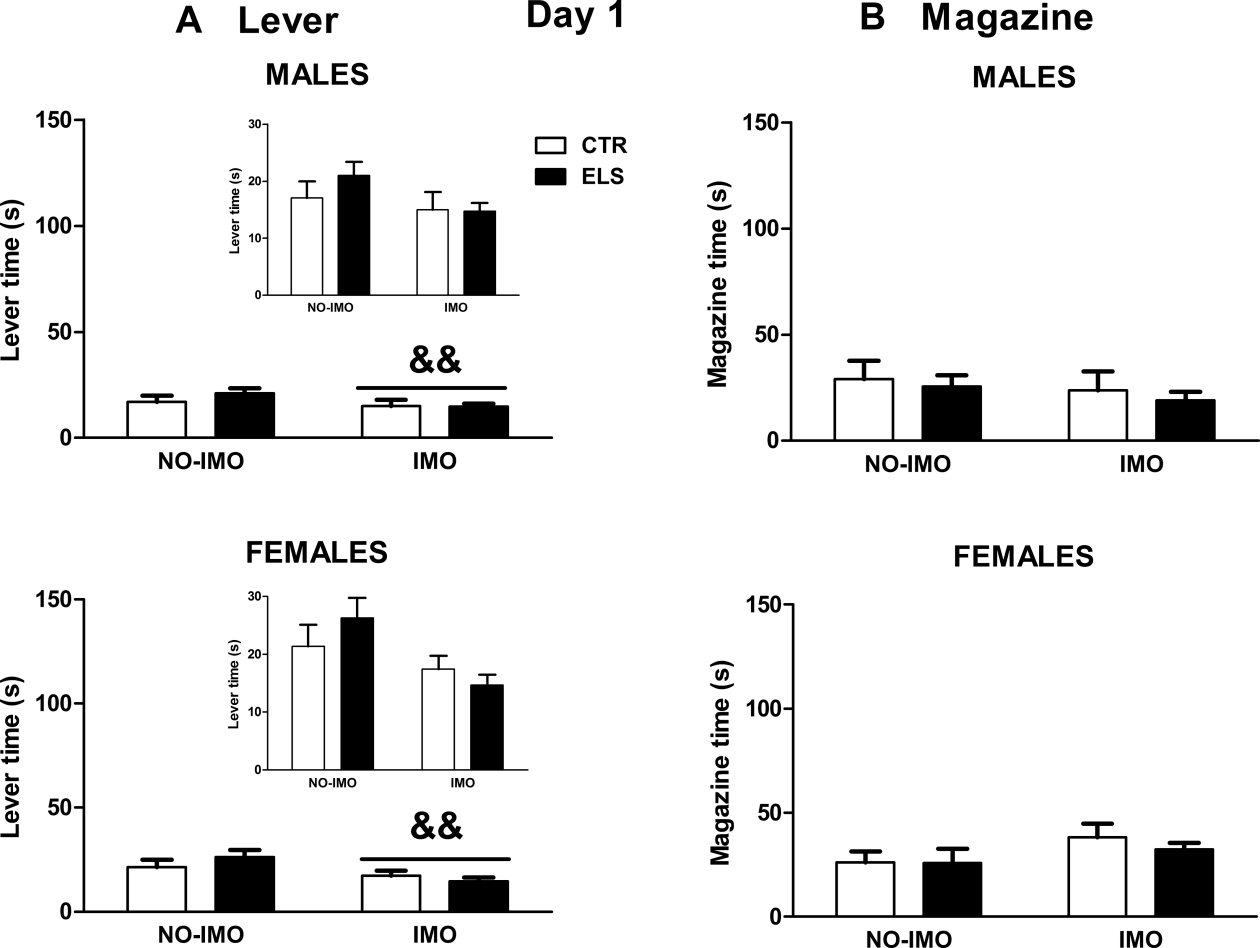
Sign-tracking and goal-tracking behavior in day 1. Time (s) spent in behavior towards the lever (left panels) and time (s) spent inside the magazine during lever presentation (right panels) for day 1. Results are shown for control (CTR) and early-life stress (ELS) rats of both sexes, exposed to immobilization (IMO) stress or not when adults. && p < 0.01 versus non-IMO. The same scale as in Fig 5 is maintained to allow comparisons between days 1 and 5. In the insets, the same results with different scale are provided. Means and SEM are shown.

The time spent in behavior towards the lever and inside the food magazine was measured for days 1 and 5. On day 1 (Fig 4A), the statistical analysis showed that the impact of IMO was statistically significant for time spent in behavior towards the lever [F(1,69) = 10.1, p < 0.01). IMO by itself induced a reduction in time spent in contact with the lever. On that day, no statistically significant differences were found in time inside the magazine either during lever presentation (Fig 4B) or entries during the ITI and pellet presentation [SEX, ELS and IMO, and all interactions: NS, in all cases].

**Fig 5:**
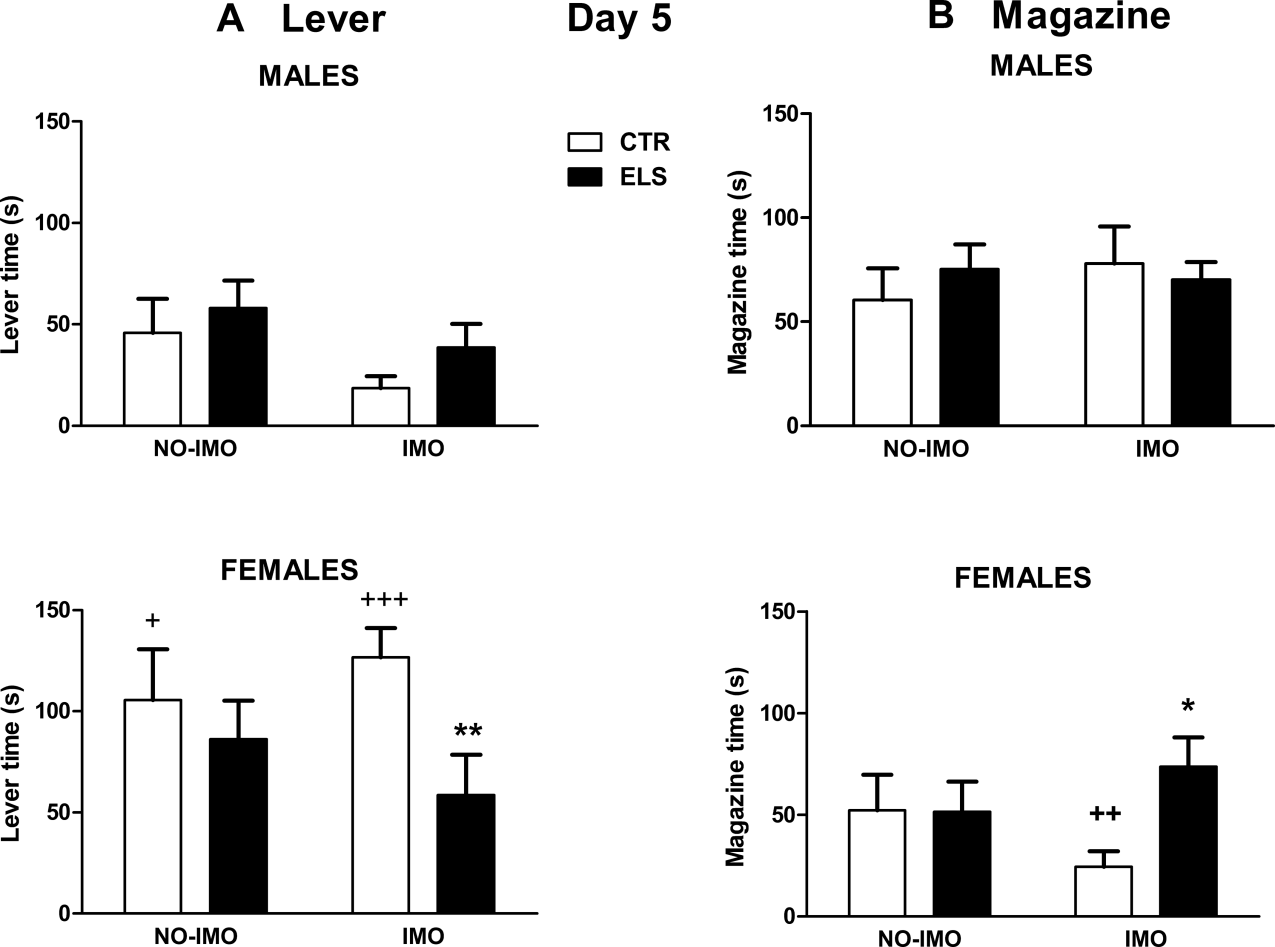
Sign-tracking and goal-tracking behavior in day 5. Time (s) in behavior towards the lever (left panels) and time (s) spent inside the magazine during lever presentation (right panels) for day 5. Results are shown for control (CTR) and early-life stress (ELS) rats of both sexes, exposed or not to immobilization (IMO) stress when adults. ** p < 0.01 versus corresponding CTR; + p < 0.05, ++ p < 0.01, +++ p < 0.001 versus corresponding males. Means and SEM are shown.

After training, the statistical analysis of time spent in behavior towards the lever on day 5 (Fig 5A) indicated that only SEX and the interaction SEX x ELS were statistically significant [X^2^(1) = 22.16, p < 0.001; X^2^(1) = 6.78, p < 0.01, respectively]. The planned contrast in IMO-exposed rats showed that the ELS factor was significant only in females (p < 0.01) and that the SEX factor was significant only in animals not exposed to ELS (p < 0.001). Thus, after IMO, control females approached/interacted more with the lever than males, and exposure to the early treatment decreased behavior towards the lever only in females. In the animals neither exposed to IMO nor to ELS, the SEX factor was also statistically significant, with females spending more time in behavior towards the lever (p < 0.05). In an additional analysis, “preparatory” and “consummatory” behavior towards the lever was differentiated, and the statistical analysis showed that the reported differences between groups in the global measure were due to “consummatory” and not “preparatory” behavior. Orienting behavior towards the lever on day 5 was very low.

**Fig 6:**
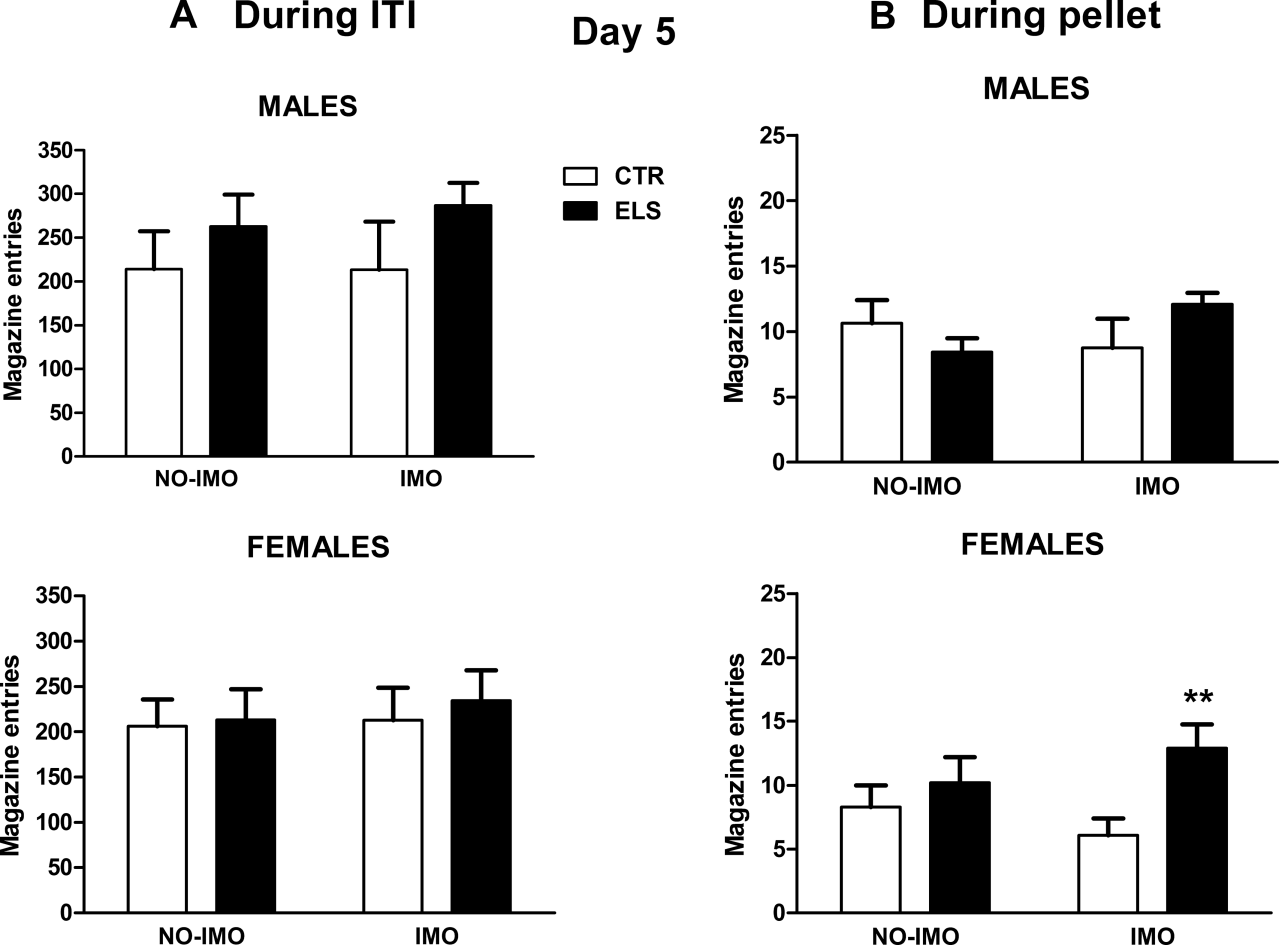
Magazine entries. Magazine entries during the ITI (left panels) and magazine entries during pellet delivery (right panels) for day 5. Results are shown for control (CTR) and early-life stress (ELS) rats of both sexes, exposed to immobilization (IMO) stress or not when adults. * p < 0.05 versus corresponding CTR. Means and SEM are shown.

Regarding time spent inside the magazine during lever presentation on day 5 (Fig 5B), SEX was the only statistically significant factor [F(1,72) = 4.60, p < 0.05], whereas the interaction SEX x ELS x IMO approached significance [F(1,72) = 3.59, p = 0.06]. The planned comparison in IMO-exposed animals indicated that ELS increased magazine time during lever presentation only in females (p < 0.05) and that non-ELS females spent less time inside the magazine than corresponding males (p < 0.01). Magazine entries during the ITI did not differ between groups (all factors and interactions: NS, Fig 6A). Regarding the number of entries inside the magazine during pellet presentation (Fig 6B), the statistical analysis indicated that ELS [F(1,72) = 4.74, p < 0.05] and ELS x IMO [F(1,72) = 5.36, p < 0.05] factors were statistically significant. The planned contrast in IMO-treated animals showed that ELS increased entries during pellet presentation only in females (p < 0.01). Moreover, ELS decreased the latency to approach the magazine after lever presentation, regardless of SEX and IMO [F(1,72) = 4.34, p < 0.05, all the other factors and interactions: NS].

**Fig 7:**
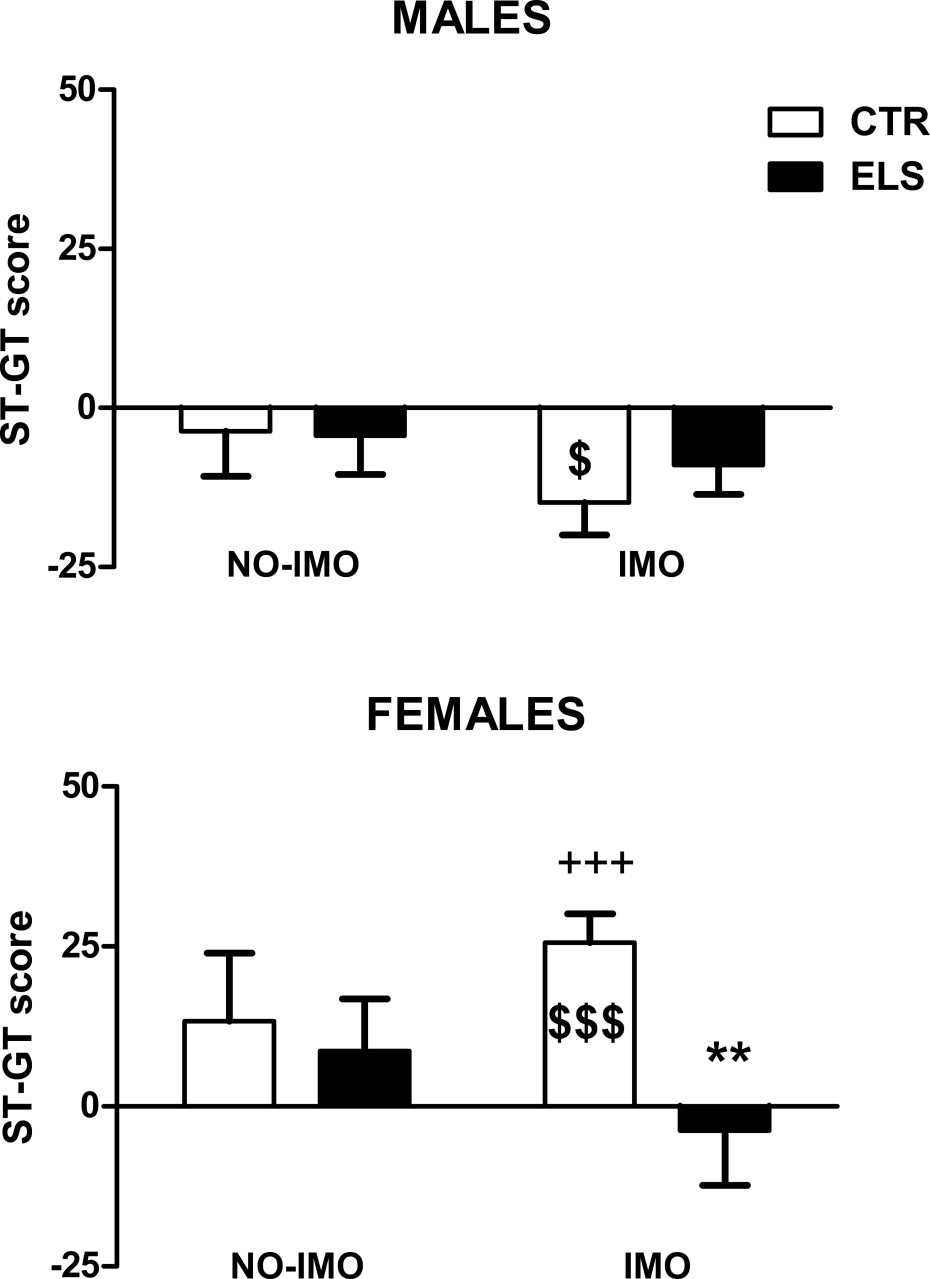
Sign-tracking (ST)–Goal-tracking (GT) score for day 5. A positive score indicates that ST behavior predominates over GT, whereas a negative score indicates that GT behavior predominates over ST. The results are shown for control (CTR) and early-life stress (ELS) rats of both sexes, exposed to immobilization (IMO) stress or not when adults. ** p < 0.01 versus corresponding CTR; +++ p < 0.001 versus corresponding males. When compared against a “zero” value, the score was only statistically significant for IMO males and females not exposed to ELS ($ p < 0.05; &&& p < 0.001). Means and SEM are shown.

The statistical analysis of the ST-GT score on day 5 (Fig 7) indicated that SEX and the interaction SEX x ELS were statistically significant [X^2^(1) = 15.21, p < 0.001; X^2^(1) = 4.11, p < 0.05, respectively], and the other factors and interactions were NS. However, the planned contrast in the IMO-treated rats showed that ELS decreased this score in females (p < 0.01) and not in males and that female control rats (non-exposed to ELS) had a higher index than males (p < 0.001). When the ST-GT score was compared against “zero”, the only subgroup that had clearly GT behaviors were non-ELS males exposed to IMO (p < 0.05), whereas females of the same treatment were the only ones that performed ST clearly (p < 0.001). When only the subjects selected for histological analysis were studied (n = 64), the interaction SEX x ELS X IMO was statistically significant [X^2^(1) = 3.96, p < 0.05], and the two above mentioned differences were maintained.

**Fig 8:**
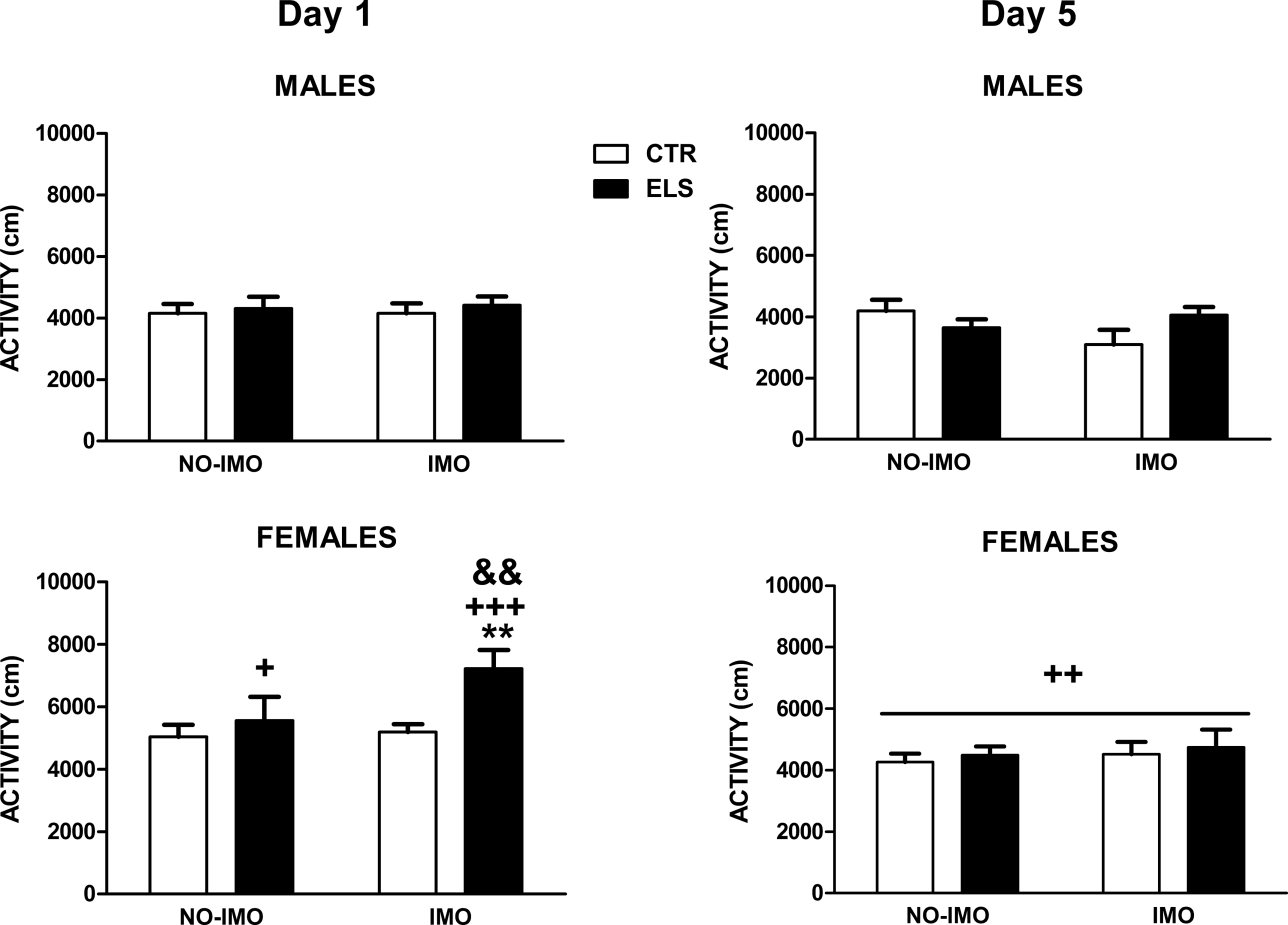
Motor activity (cm) inside the chamber for day 1 and 5. Results are shown for control (CTR) and early-life stress (ELS) rats of both sexes, exposed to immobilization (IMO) stress or not when adults. ** p < 0.01 versus corresponding CTR; + p < 0.05, ++ p < 0.01, +++ p < 0.001 versus corresponding males; && p < 0.01 versus corresponding non-IMO. Means and SEM are shown.

Motor activity inside the operant chamber was measured on days 1 and 5 (Fig 8). For day 1, the statistical analysis showed that SEX [F(1, 71) = 21.62, p < 0.001] and ELS [F(1, 71) = 5.32, p < 0.05] were statistically significant, and the other factors and interactions were NS. However, the planned contrast in the IMO-treated rats showed that (i) ELS increased motor activity in females (p < 0.01), and not in males, and (ii) IMO females exposed to ELS were more active than the corresponding males (p < 0.001) and than non-IMO-treated females (p < 0.01). In the animals not exposed to IMO, the SEX effect was only statistically significant in ELS animals (p < 0.05). For day 5, the ANOVA indicated that the only statistically significant factor was SEX [F(1,72) = 8.32, p < 0.01], with females more active than males.

### Dopaminergic markers

**Fig 9:**
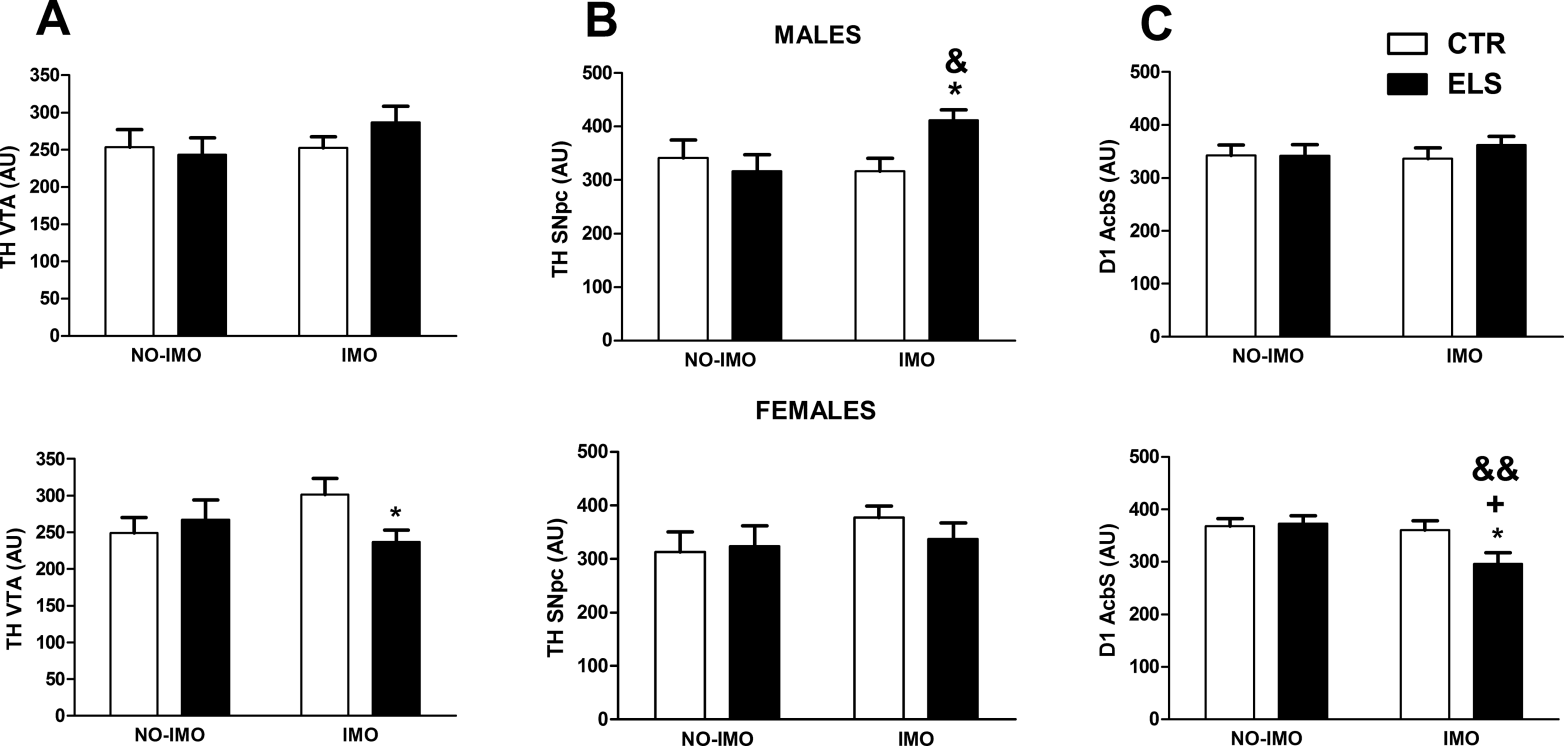
Expression of dopaminergic markers. Tyrosine hydroxylase (TH) expression in the ventral tegmental area (VTA) and the substantia nigra pars compacta (SNpc) and D1 dopamine receptors in the nucleus accumbens shell (AcbS) in arbitrary units (AU). The results are shown for control (CTR) and early-life stress (ELS) rats of both sexes, exposed to immobilization (IMO) stress or not when adults. * p < 0.05 versus corresponding CTR; + p < 0.05 versus corresponding males; & p < 0.05, && p < 0.01 versus corresponding non-IMO. Means and SEM are shown.

The statistical analysis of the TH expression in the VTA (Fig 9A) indicated that the interaction SEX x ELS x IMO was statistically significant [F(1,55) = 4.46, p < 0.05], and the other factors and interactions were NS. The decomposition of the interaction indicated that, only in IMO-females, ELS decreased TH expression (p < 0.05). Regarding TH expression in the SNpc (Fig 9B), the interaction SEX x ELS x IMO was also statistically significant [F(1,56) = 4.02, p = 0.05], and the other factors and interactions were NS. The decomposition of the interaction indicated that prior ELS increased TH expression in males exposed to IMO in comparison to non-ELS exposed males (p < 0.05) and males not exposed to IMO (p < 0.05). On the other hand, D1 expression in the AcbS (Fig 9C) paralleled some changes in TH expression in the VTA. Although the interaction SEX x ELS x IMO only approached significance [F(1,56) = 3.45, p = 0.068], and the other factors and interactions were NS. The decomposition of the interaction showed that in IMO-exposed females exposed to ELS decreased D1 expression in comparison to non-ELS exposed females (p < 0.05), to ELS males (p < 0.05) and to non-IMO exposed females (p < 0.01).

Finally, with regard to the expression of TH in LC and expression of D1 in DLST and DMST, no statistically significant differences between groups were observed (S1 Table). Representative photographs of TH and D1 expression are shown in S2–S6 Figs.

Behavioral data did not correlate to ISH expression in any brain area analyzed. Several statistically significant positive correlations were found between TH expression [TH in VTA with TH in SNpc: r (61) = + 0.74, p < 0.001; TH in LC with TH in SNpc: r (62) = + 0.47, p < 0.001; TH in LC with TH in VTA: r(61) = + 0.46, p < 0.001] and between D1 expression [D1 in DLST with D1 in DMST: r(62) = + 0.96, p < 0.001; D1 in DLST with D1 in AcbS: r (62) = + 0.89, p < 0.001; D1 in DMST with D1 in AcbS: r(62) = + 0.89, p < 0.001] in different areas. However the correlations between D1 and TH expression were lower [D1 in DMST with TH in LC: r(62) = + 0.32, p < 0.05; D1 in AcbS with TH in VTA: r(61) = + 0.28, p < 0.05; D1 in AcbS with TH in LC: r(61) = + 0.30, p < 0.05].

## Discussion

The present study indicates that the attribution of incentive salience to food-associated cues was affected by early-life and adult stressors in a sex-dependent manner. In control animals, females presented slightly greater ST behavior than males. Our initial hypothesis that exposure to IMO would decrease ST behavior, especially in females, was not confirmed because it was males who showed reduced ST behavior after IMO. As measured by the ST-GT score, the only animals that showed clear GT behavior were males exposed to IMO, whereas females exposed to IMO presented clear ST behavior. In IMO females, ELS was able to reduce the ST-GT score. This decrease in the attribution of incentive salience was accompanied by a reduction in TH expression in the VTA and D1 expression in AcbS. The ELS treatment by itself was ineffective. The functional implications and specificity of these findings are discussed below.

### Sex differences in the attribution of incentive salience

In absolute control animals (neither exposed to ELS nor to IMO) after the conditioning procedure (day 5), ST behavior was slightly higher in females than in males (Fig 5A), indicating that the attribution of incentive salience in rats may be sex-dependent. GT behavior (as measured by time spent inside the magazine during lever presentation) was not affected by sex, and magazine entries during the ITI were also the same in females and males, as was latency to approach the magazine (once the lever was inserted). However, the proper ST-GT score (day 5) was either not significantly different between male and female control animals (not exposed to any stress), indicating that the sex differences detected were modest.

Recent studies have addressed the role of sex differences in incentive salience attribution. The presence of sex-related differences in conditioned approach behavior is in line with other studies where female rats acquired ST behavior slightly faster than males [47, 54] or presented higher ST behavior than males sustained in time [55]. However, sex differences in ST and GT behavior may be strain-dependent since they have not been detected in all studies [56]. Although we cannot rule out that the present subtle sex differences are more related to an increase in exploration as a consequence of an unexpected/salient stimulus (lever extension), the apparent slightly higher degree of incentive salience to reward-related cues detected in females is in line with the fact that females are more vulnerable to different aspects of addictive behavior than males [57].

### The effects of exposure to stress in the attribution of incentive salience

Previous studies have shown that early life experiences exert important long-term effects in responsiveness to reward-related cues. Male rats reared without a mother (“artificially” reared rats) [58] or exposed to ethanol during adolescence [59] showed an increase in adult ST behavior, whereas environmental enrichment after weaning reduces it [60]. In our conditions, early stress was ineffective per se (without IMO treatment) in both males and females. To our knowledge, no previous data have addressed the effects of adult traumatic stressors on the attribution of incentive salience and the interaction between early stress and the exposure to another stressor during adulthood. In rats not exposed to early stress but exposed to IMO, ST behavior (time spent in behavior towards the lever) was higher in females than males. In addition, in those animals, GT (time spent inside the food-magazine when the lever was present) was lower in females than males. When comparing behavior towards the lever on days 1 and 5, non-ELS males exposed to IMO do not develop ST behavior with training. The ST-GT score indicated that non-ELS males exposed to IMO were more GT than ST, whereas the opposite was observed in females (more ST than GT). Prior ELS seems to “normalize” the extreme sex-dependent phenotypes induced by IMO. The impairment in the development of ST behavior seen in males after IMO fits well with human data where PTSD patients present a blunted response to reward-related cues [11], although we expected an even more blunted response to reward in females based on human data [12, 13]. The possible reasons for these discrepancies with human data are unclear, but several factors may account for them, such as the presence of different type/intensity of traumatic stressors between male and female humans (instead of, or in addition to, different biological vulnerability between sexes) or the translational value of the animal models used.

The ELS used in the present study (restriction of nesting material) in our hands induced an increase in the maternal care received, as measured by arched-back and licking-grooming behavior. These data agree with our previous work using a similar model [41], but appear to be in contrast with other results [61]. Although in other studies the restriction of nesting material did not change arched-back or licking-grooming behavior [62], the ability to develop adequate maternal care in absence of nesting material varies between strains [63, 64].

Importantly, females seem to be more sensitive to the long-term effects of ELS in that exposure to IMO after prior ELS clearly decreased ST (behavior towards the lever) and increased GT (behavior towards the magazine). It is of note that females also seem more sensitive to the immediate impact of the ELS, as measured by decreased body weight, a marker of the intensity of the stressors [16]. Although individual maternal behavior for each pup was not measured, it has been described that male pups receive more maternal care than females [9, 65]. It has been shown that maternal care may “buffer” some of the negative sequels of stress [41–43], the impact of ELS should be globally higher in females than males because compensatory attention to pups was probably more directed to males than females. Assuming that the intensity of the ELS was higher in females, it appears that this treatment was able to reduce the impact of adult IMO on ST behavior. As excessive ST is considered a non-adaptive behavior (see below for possible functional implications of ST/GT behavior) and thus was reduced by previous ELS, globally these data fit better with the “match-mismatch” conceptualization that proposes that a previous experience with stress may favor coping towards further stressors (e.g., [66–68]). However, the functional impact of ST/GT behavior is still an open question, as will be mentioned later.

Are the differences in ST/GT behavior (day 5) specifically related to the conditioning procedure? On the first day of autoshaping training, all the groups approached the magazine to the same extent during lever presentation, suggesting that the motivational value of the food itself was not modified by the treatments. Magazine entries during the ITI (instead of lever presentation) was not affected by the treatments (day 1 and 5). However, IMO-exposed animals (regardless of sex and early treatment) spent less time in behavior towards the lever on day 1, suggesting that IMO induced a transient reduction of interest for a novel/unexpected stimulus. This possible lack of interest in the lever at the beginning of the procedure was not accompanied by a general decrease in motor activity (as discussed later).

General motor activity inside the chamber at the beginning of the procedure (day 1) was higher in females exposed to ELS plus IMO in comparison to the other groups. The impact of ELS plus IMO vanished by day 5, where general activity inside the chamber was only affected by sex (females being all more active than males). Although it cannot be ignored that initially higher levels of activity/exploration were responsible for some of the observed changes in ST/GT behavior on day 5, our data suggest that may be dissociated. Motor activity on day 1 did not correlate with time spent inside the magazine during lever presentation on day 5, but in the whole population it modestly correlated positively (and not negatively) with time spent in behavior towards the lever on day 5 [r(78) = + 0.24, p < 0.05]. Taken together, the results suggest that the specific decrease in ST induced by ELS combined with IMO in females does not seem to be attributed to pre-existing differences before training or to a decrease in general activity/exploration.

### The role of dopamine in sign-tracking and goal-tracking behavior

Dopamine is involved in several appetitive and aversive motivational processes that include learning subjective reward value, exertion of effort, behavioral activation, maintenance of sustained task engagement and attribution of incentive salience to reward-associated cues [69–71]. The cerebral circuits involved in incentive salience attribution are complex. Several brain structures that receive dopaminergic projections seem implicated in ST and/or GT behavior, such as AcbS [72], dorsomedial striatum [73], dorsolateral striatum [74], or medial prefrontal cortex [75]. The precise dopaminergic profile of GT versus ST animals has not been fully characterized. Initial data indicated that, after training, GT animals have a greater expression of TH and a dopamine transporter in the VTA in comparison to ST animals, with no changes in D1 expression in the Acb, as measured by in situ hybridization [35]. In other studies, GT rats had lower dopamine transporter surface expression in synaptosomes from the ventral striatum in comparison to ST rats accompanied by a slower dopamine uptake [36]. In addition, ST behavior seems to be more sensitive to dopaminergic modulation than GT behavior [37–39]. It has to be noticed that in other studies the intermediate phenotypes between clear ST and GT behavior are not included in the analysis, representing thus a different phenotype of animals than the ones used in the present work where all the subjects are included.

In the present study, the decrease in ST behavior observed in ELS females exposed to IMO was accompanied by decreased TH expression in the VTA and in D1 expression in the AcbS but not in the other brain areas analyzed. A remaining question to be addressed in future studies is whether the observed changes in TH and D1 receptor expression are pre-task traits or they were induced by behavioral training.

TH is the rate-limiting enzyme for dopamine synthesis. It is assumed that there is some sexual dimorphism in midbrain dopaminergic systems in basal conditions and in response to drugs and stress [76]. Moreover, previous studies have found that TH levels (in VTA and SNpc, as measured by immunohistochemistry) are very sensitive to early life interventions in a developmental and sex-dependent manner (e.g., [77]). The functional meaning of a reduction of TH levels in the VTA is not well-established. Although in some studies a decrease in these levels were obtained in prolonged withdrawal after chronic cocaine treatment [78], probably related to the anhedonic state induced, this decrease has not been obtained in all the studies (see [79] for a discussion). In contrast to the reduction of TH VTA levels induced by the early stress treatment plus IMO in females, in males, the effects of the same treatment were only detected in the SNpc and were of opposite sign. In males exposed to IMO, the early stress treatment increased (not decreased) the expression of TH in the SNpc.

The reduction in D1 expression in the AcbS detected in ELS females submitted to IMO is in line with a decrease in the motivational impact of reward-associated cues. Previous studies found that microinjection of D1 antagonists into the AcbS reduced cocaine-primed reinstatement [80, 81] and context-induced reinstatement of heroin [82, 83], morphine [84] and ethanol [85]. Regarding the Pavlovian instrumental transfer phenomena (another measure of incentive salience attribution), previous data indicated that it was largely absent in animals that received the intra-AcbS microinjection of a D1 antagonist [86, 87]. However, the specific role of D1 receptors in AcbS in incentive salience is unclear, as virus driven D1 expression in the AcbS of D1 KO mice improves instrumental responding for reward but not Pavlovian conditioned responding [88]. Considering that we did not observe correlations between the dopaminergic markers analyzed and behavior, it is possible that neurochemical and behavioral effects were unrelated. Nevertheless, the neurochemical data here reported might be considered as preliminary and exploratory. A relationship might appear using more in depth characterization of the dopaminergic system, including the expression of other dopaminergic markers in areas such as the medial prefrontal cortex or experiments blocking D1 receptors in the latter areas and in striatal regions.

### Functional implications of sign-tracking and goal-tracking behavior

Individual differences in the attribution of incentive salience to reward-related cues in humans [32, 33] and animals [28, 31] may underlie different adaptive learning styles (different strategies of learning), but in extreme cases may lead to maladaptive behavior and psychopathology. There are important differences in the development of ST behavior between strains of rats [89, 90] and even between vendors of the same strain [91]. A clear distinction between predominantly ST and GT behavior has not been obtained in previous studies (e.g., [92]). In the present study, most of the animals showed an intermediate phenotype between extreme ST and GT behavior, except for the IMO-treated rats not exposed to ELS. Male IMO-exposed animals were overtly more GT than ST, whereas female IMO-exposed animals were more ST than GT.

What are the functional consequences of being ST versus GT? Since the initial proposal that the sensitization of incentive salience to drugs and drug-associated stimuli is a key factor in the development of addictive behavior [93], several available data indicate that those individuals for whom Pavlovian reward cues are powerful incentives are in general more vulnerable to different impulse control diseases (see [94] for a review). The exaggerated attribution of incentive salience to cues paired with food has been related to impulsive action but not impulsive choice [55, 87, 95], novelty-seeking [87, 96], behavioral inflexibility [97] or poor attentional control [98]. ST rats also have better ability to associate specific (tone) cues that predict footshock (cued fear conditioning) versus GT rats, which are more vulnerable to developing contextual fear conditioning [99]. In other studies, ST animals developed an auditory fear conditioning to the same extent as GT rats but presented more fear incubation [100]. The differences in the ability to develop/express cued (auditory) versus contextual Pavlovian conditioning points to the discussion of whether these two types of extreme populations may represent different pathways to disease rather than a differential vulnerability *per se* [33]. Although most of the available data strengths, the “negative” value of an excessive ST behavior, an inadequate/low attribution of incentive salience may also be related to the development of anhedonic-like behavior and psychopathology.

The results showed that the interaction between sex and early and adult stressors was more complex than predicted. Exposure to IMO seems to decrease ST behavior to food-related cues in males, and has the opposite effect in females. The impact of IMO in females is reduced by previous ELS, accompanied by changes in key dopaminergic changes. Thus, in agreement with our previous data, the impact of both ELS [41] and of severe stressors such as IMO [24] are sex-dependent. Our data globally strengthen the finding that sex is an important factor in the study of the effects of stress in animal models of neuropsychiatric diseases [101, 102].

## Supporting information

S1 Fig Localization of regions analyzed. Adapted from [49]. In A: 1: dorsomedial striatum (DMST); 2: dorsolateral striatum (DLST); 3: accumbens shell (AcbS). In B: 4: substantia nigra pars reticulata (SNpc); 5: ventral tegmental area (VTA). In C: 6: locus coeruleus (LC).
(TIF)

S2 Fig Representative autoradiographs of tyrosine hydroxylase in the ventral tegmental area. Representative autoradiographs of tyrosine hydroxylase (TH) mRNA in the ventral tegmental area (VTA) of male (top) and female (bottom) rats exposed or not to early-life stress (ELS) and exposed to immobilization (IMO) or not in adulthood. The area where the measurements were made is highlighted.
(ZIP)

S3 Fig Representative autoradiographs of tyrosine hydroxylase in the substantia nigra pars compacta. Representative autoradiographs of tyrosine hydroxylase (TH) mRNA in the substantia nigra pars compacta (SNpc) of male (top) and female (bottom) rats exposed to early-life stress (ELS) or not and exposed to immobilization (IMO) or not in adulthood. The area where the measurements were made is highlighted.
(ZIP)

S4 Fig Representative autoradiographs of tyrosine hydroxylase in the locus coeruleus. Representative autoradiographs of tyrosine hydroxylase (TH) mRNA in the locus coeruleus (LC) of male (top) and female (bottom) rats exposed to early-life stress (ELS) or not and exposed to immobilization (IMO) or not in adulthood. The area where the measurements were made is highlighted.
(ZIP)

S5 Fig Representative autoradiographs of dopamine 1 receptors in the dorsomedial and dorsolateral striatum. Representative autoradiographs of dopamine 1 receptors (D1R) mRNA in the dorsomedial (DMST) and dorsolateral (DLST) striatum of male (top) and female (bottom) rats exposed to early-life stress (ELS) or not and exposed to immobilization (IMO) or not in adulthood. The area where the measurements were made is highlighted.
(TIF)

S6 Fig Representative autoradiographs of dopamine 1 receptors in nucleus accumbens shell. Representative autoradiographs of dopamine 1 receptors (D1R) mRNA in nucleus accumbens shell (AcbS) of male (top) and female (bottom) rats exposed to early-life stress (ELS) or not and exposed to immobilization (IMO) or not in adulthood. The area where the measurements were made is highlighted.
(ZIP)

S1 Table Other dopaminergic markers. mRNA levels of tyrosine-hydroxylase (TH) in the locus coeruleus (LC) and dopamine D1 receptors in the dorsolateral striatum (DLST) and the dorsomedial striatum (DMST). CTR: control; ELS: early life stress; IMO: immobilization.
(PDF)

S1 File Raw data. Individual data for each animal of the measures analyzed.
(XLSX)
