# Supplementary figures and images for: Sex-dependent impact of early-life stress and adult immobilization in the attribution of incentive salience in rats
Source: PLoS One. 2018 Jan 11;13(1):e0190044. doi: 10.1371/journal.pone.0190044 (PMC5764258; doi:10.1371/journal.pone.0190044)

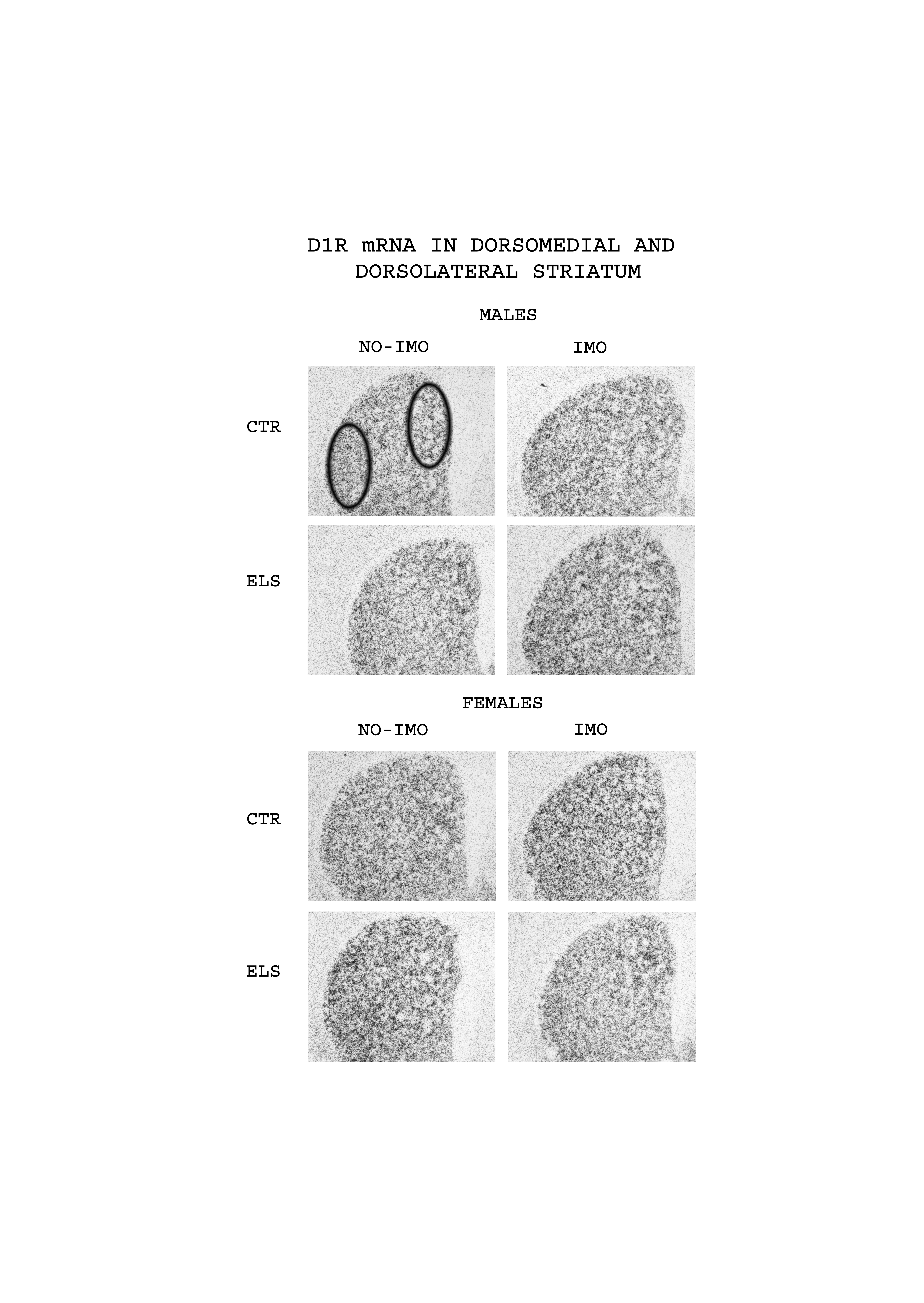

Supplement: S5 Fig — Representative autoradiographs of dopamine 1 receptors (D1R) mRNA in the dorsomedial (DMST) and dorsolateral (DLST) striatum of male (top) and female (bottom) rats exposed to early-life stress (ELS) or not and exposed to immobilization (IMO) or not in adulthood. The area where the measurements were made is highlighted. (TIF) [file pone.0190044.s005.tif]
