# Supplementary material for: Sex-dependent impact of early-life stress and adult immobilization in the attribution of incentive salience in rats
Source: PLoS One. 2018 Jan 11;13(1):e0190044. doi: 10.1371/journal.pone.0190044 (PMC5764258; doi:10.1371/journal.pone.0190044)
Supplement: S1 Table — mRNA levels of tyrosine-hydroxylase (TH) in the locus coeruleus (LC) and dopamine D1 receptors in the dorsolateral striatum (DLST) and the dorsomedial striatum (DMST). CTR: control; ELS: early life stress; IMO: immobilization. (PDF) [file pone.0190044.s007.pdf]

Supplementary Table 1

SEX	EARLY LIFE TREATMENT	ADULT TREATMENT	mRNA TH	mRNA D1	
			LC	DLST	DMST
MALE	*CTR*	*NO-IMO*	753 ± 65	385 ± 22	390 ± 20
		IMO	719 ± 47	386 ± 21	393 ± 18
	ELS	*NO-IMO*	712 ± 58	386 ± 25	387 ± 19
		IMO	782 ± 58	420 ± 24	419 ± 23
FEMALE	*CTR*	*NO-IMO*	645 ± 50	426 ± 17	425 ± 13
		IMO	829 ± 58	404 ± 20	403 ± 18
	ELS	*NO-IMO*	641 ± 82	404 ± 22	407 ± 20
		IMO	667 ± 51	347 ± 25	349 ± 24

mRNA levels of tyrosine-hydroxylase (TH) in the locus coeruleus (LC) and dopamine D1 receptors in the dorsolateral striatum (DLST) and the dorsomedial striatum (DMST).

CTR: control; ELS: early life stress; IMO: immobilization
